# Emotional, coping factors and personality traits that influenced alcohol consumption in Romanian students during the COVID-19 pandemic. A cross-sectional study

**DOI:** 10.1186/s12889-024-18247-w

**Published:** 2024-03-07

**Authors:** Cornelia Rada, Cristina Faludi, Mihaela Lungu

**Affiliations:** 1 Biomedical Department, Francisc I. Rainer Institute of Anthropology, Romanian Academy, Academy House 13 September Avenue, No. 13, 5th District, 050711 Bucharest, Romania; 2 Faculty of Sociology and Social Work, Social Work Department, Babeș-Bolyai University, Cluj-Napoca, Romania; 3 Argeș County Centre for Educational Resources and Assistance, Pitești, Romania

**Keywords:** Alcohol consumption, Personality, Depression, Anxiety, Antisocial actions, COVID-19 pandemic, Aggressiveness, Somatic complaints

## Abstract

**Background:**

During the COVID-19 pandemic, after 3 months from the installation of the state of emergency on the territory of Romania, data were collected from 677 students and master’s students, to explore the problematic alcohol consumption (AC).

**Methods:**

The evaluation was done with: Alcohol Use Disorders Identification Test, Depression, Anxiety and Stress Scales, Strategic Coping Approach Scale and The Freiburg Personality Inventory. The statistical methods used were linear regression with bootstrap procedure, Spearman’s rank correlation, and the Mann-Whitney U test.

**Results:**

More than half were affected by depression or anxiety of moderate to extremely severe intensity. The prevalence of problematic alcohol consumption was low: (Hazardous and Extremely Hazardous (2.3) and Medium Risk (10.2). Early onset increases the subsequent risk of problematic AC, compared to women, men recorded a higher AC (*p* <.01). Anxiety, antisocial action, personality traits Aggressiveness and Somatic complaints had the effect of increasing the alcohol consumption score (*p* <.01). Significant but weak positive correlations between AC on one hand, and depression, anxiety, stress and antisocial action on the other hand were found (*p* <.01).

**Conclusions:**

Probably the prevalence of AC was low as a result of the fact that most respondents were studying in the field of health promotion and as a result of the closure of entertainment venues. This study advocates for the education of youngsters to clearly express their opinions without violating the boundaries of others’ feelings (assertive action) and to act prudently in dangerous or uncertain situations (cautious action) since these coping mechanisms were not associated with problematic alcohol consumption. The promotion of positive, achievement-oriented, life attitudes is equally important, as these characteristics of the Life Satisfaction personality dimensions were also found as non-determinants of alcohol-induced problems. The association of problematic AC with antisocial actions as a coping mechanism and high scores on Aggressiveness calls for interventions to educate the younger generation how to acquire and adopt healthy mechanisms to control tensions without resorting to alcohol consumption, more so as the two variables reinforce each other. Drinking as a means of gaining courage must be carefully reconsidered since anxiety generally hits back, often in increased levels.

## Background

Concerns about the social implications of the COVID-19 pandemic are a topical issue in spite of the huge number of related studies published since its outbreak. The pandemic can be viewed as a prolongued global trauma that generated serious health, social and economic consequences among a plethora of negative emotions.

The imposed social distancing measures generated exacerbated feelings of loneliness and exclusion able to trigger aggressive reactions for mere social media mishaps whenever the elementary need of belonging was violated. Uncertainties related to infection, treatment and subsequent recovery, the bluring predictability of the bumpy course towards reinstation of normal life, could have led to aggressive behaviors during the pandemic. Prolongued home entrapment preventing people’s access to their workplaces (school included) proved to be particularly frustrating.

Studies have shown an increased aggression during the COVID-19 lockdowns [1, 2], as well as an escaladation of domestic violence [3]. In their study on 815 middle aged subjects from Kuwait during the COVID-19 pandemic, Al-Sejari & Al-Ma’seb [4] profiled the persons more likely to frequently report instances of physical and verbal aggression, as well as hostility: males, younger persons, single or divorced status, students, individuals with a lower level of education.

The limitation of outdoor activities had harmful effects on physical and mental health for schoolchildren and students in particular, more so for those in boarding schools [5]. While the limitation of social contacts and outdoor activities during the pandemic induced a feeling of safety because of decreased contamination risks, isolation from family or friends generated negative emotions such as sadness, anxiety, irritability or stress [6–8].

A number of studies have indicated a significant deterioration of mental health during the pandemic [1, 9] often associated with alcohol consumption [10].

The pandemic generated high levels of stress, justifiably associated with increased alcohol consumption. However, certain studies have argued that such association was far from being simplistic. Jaffe et al. [11] surveyed 694 students from an important public university in Northwestern US between November 2019 and September 2021 and found that stress was associated with increased alcohol consumption in those lacking a meaningful life.

Studies on the problematic alcohol consumption (PAC) during the COVID-19 pandemic produced varied results. In contrast to Schecke et al. [12], which found increased alcohol consumption (AC) levels in subjects already accustumed to heavy drinking, Calina et al. [13] observed a PAC decrease, while Rada & Lungu [14] noted no changes compared to pre-pandemic periods.

Such differences may be due to geographical and cultural specifics of various populations, age groups, the tools employed in the process or the data collection period in reference to the moment the restrictions were imposed.

Likewise, increases in interpersonal violence, self-aggression [15] in those dramatic periods require an appropriate approach in order to identify the triggers leading to alterated personality traits and decipher the coping mechanisms involved.

Several questions arose: What defense mechanisms did people appeal to during the pandemic in order to counter negative emotions? Was desensitization a justification for alcohol consumption? Which personality traits could predict a better or worse adaptation to crisis?

Although the pandemic has passed exploring the lessons learnt during the COVID-19 pandemic is important in teaching children and young people how to cope when faced with stress or negative emotions.

Few studies have simultaneously addressed the influence of negative emotions, coping mechanisms and personality traits in conjunction with PAC during the pandemic.

This study aims to increase knowledge about AC among Romanian students following the first of the five COVID-19 infection waves. The research questions are:

- Is there a connection between PAC and negative emotions experienced during the pandemic?
- Did behavioral coping influence the drinking problems?
- What personality dimensions were involved in PAC.

The main objective of this study was to identify the relationships between alcohol consumption on one hand, and emotional states of depression, anxiety and stress, behavioral coping dimensions and personality traits on the other.

The research hypothesis assumed that emotional states, the behavioral dimensions of coping, the personality traits, age and gender can be significant predictors of alcohol consumption.

## Methods

### Research context

Taking into consideration the evolution of the international epidemiological situation determined by the spread of the SARS-CoV-2 coronavirus, a state of emergency was decreed in Romania on March 16, 2020 and extended on April 14, 2020. A state of alert was later imposed through a series of laws and ordinances implementing prevention measures similar to those other European Union countries took to contain the spreading of the coronavirus. The state of alert was finally lifted on March 8, 2022, when the fifth wave of COVID-19 cases dropped [16].

The sample of this research was made up of students and master’s students at universities in Romania. The data were collected between June 2020 and November 2021, during the restrictions imposed by the SARScov-2 virus pandemic, when university courses in the classic, face-to-face format were suspended and online education was switched over.

### Sampling procedure

Teaching staff presented the study on the occasion of teaching online courses. Students and master’s students, who wanted to participate, received an editable pdf of the questionnaire set and informed consent from their teachers. It is about more than 800 students and master’s students between the ages of 18 and 31. Respondents sent the questionnaires from a private email to a research email to which only the study coordinator had access. In this way, anonymity was ensured in relation to the teachers of the class.

Optionally, each respondent had the opportunity to write their contact details. The questionnaires where omissions or misunderstandings of some answers were identified were sent back to the respondents who gave their data, and the others were eliminated. After rigourous checking, 677 undergraduate and master’s students (75.5% females) aged between 18 and 31 years were validated to participate in the study.

For statistical identification, after receiving responses, each questionnaire was coded with a unique serial number. The personal data of the respondents have been classified.

### Work protocols

All respondents took note for written consent for participation and that the European and national data processing standards are respected in each of the stages of the research. The research followed the Declaration of Helsinki to grant respect for human rights, and based on the analysis of the informed consent questionnaire and the data collection procedure, the Ethics Committee of the “Constantin Rădulescu-Motru” Institute of Philosophy and Psychology, Romanian Academy, Bucharest, approved the conduct of the research.

## Assessment Tools

Several instruments were employed in the quest of reaching the research goals. The shortened version of Lovibond’s Depression, Anxiety and Stress Scales [17], DASS– 21R, validated on the Romanian population on a non-clinical sample of 1,027 people (Perțe, coord. Albu, 2011), was employed to explore the related negative emotional states of depression, anxiety and stress. The instrument combines 7 items from each subscale: depression (α = 0.678), anxiety (α = 0.830, if the last item is removed), and stress (α =. 798). It should be noted that the DASS-21R is not used to assess current emotional states (i.e. it does not quantify how the subject feels “now”), as some items refer to experiences and situations outside the testing context.

Another tool used was the Strategic Coping Approach Scale (SCAS), validated on the Romanian population on a sample of 554 people [18]. The SCAS refers exclusively to how a person reacts behaviorally (active/passive, prosocial/antisocial, direct/indirect) following a negative experience. SCAS measures the frequency of adopting certain strategies through nine rating scales: assertive action (9 items, α = 0.611), social relationship (6 items, α = 0.605), seeking of social support (7 items, α = 0.832), prudent action (5 items, α = 0.683), instinctive action (6 items, α = 0.711), avoidance (5 items, α = 0.699), indirect action (4 items, α = 0.786), antisocial action (5 items, α = 0.792) and aggressive action (5 items, α = 0.684).

The 10 Likert-scale items in the AUDIT test developed by the World Health Organization [19] were used for a brief assessment of the alcohol consumption. The individual scores of each item were aggregated into a global quantitative indicator.

The Freiburg Personality Inventory (FPI-R, Freiburger Persönlichkeitsinventar), an omnibus assessment tool developed [20] according to the multiphasic adult model, was used to assess personality dimensions. The questionnaire was chosen because it is of a multiphase type; in the description of each scale, several adjectives correspond, which allows for a greater number of behavioral predictions. The FPI-R test was adapted and validated for Romania in 2007. The national normative reference sample for Romania included 2400 subjects equally divided by gender. For the 12 FPI-R personality measurement scales: Life satisfaction (LEB), Social Orientation (SOZ), Achievement Orientation (LEI), Inhibitedness (GEH), Excitability (ERR), Aggressiveness (AGGR), Strain (BEAN), Somatic complaints (KORP), Health Concerns (GES), Frankness (OFF), Extraversion (E), Emotionality (N) raw scores were calculated using, associated with each scale, the sum scores (0 = False or 1 = True), obtained from groups of 12 or 14 items, from the 138 items indicated in the Freiburger test methodology. The Life Satisfaction scale consist of 12 items (α = 0.514), Social Orientation consist of 12 items (α = 0.519), Achievement Orientation scale consist of 12 items (α = 0.650), Inhibitedness scale consist of 12 items (α = 0.541), Excitability scale consist of 12 items (α = 0.689), Aggressiveness scale consist of 12 items (α = 0.713), Strain scale consist of 12 items (α = 0.769), Somatic Complaints scale consist of 12 items (α = 0.753), Health Concerns scale consist of 12 items (α = 0.612), Frankness scale consist of 12 items (α = 0.662), Extraversion scale consist of 14 items (α = 0.639), and Emotionality scale consist of 14 items (α = 0.765).

## Statistical analyses

The research hypothesis assumed that emotional states, behavioral dimensions of coping, personality traits, age and gender can be significant predictors of alcohol consumption. To answer the research hypothesis, the relationship between these determinants was described by linear regression models. Statistical analyzes were conducted using SPSS 26 [21] and STATA 17 [22].

In a first regression model, emotional states and coping were analyzed in relation to alcohol consumption. The raw score for alcohol consumption served as dependent variable, while the raw scores for emotional states (depression, anxiety and stress from DASS– 21R) and the nine behavioral coping strategies of the Strategic Coping Approach Scale (SCAS) constituted the predictor variables. In a second regression model, personality traits were analyzed in relation to alcohol consumption. In this model the raw score for alcohol consumption was again used as dependent variable, while the raw scores for the 12 traits from the Freiburg Personality Inventory (FPI-R) served as predictor variables. Age and gender were additionally entered as independent variables in each of the two models.

Problems with satisfying the assumptions of heteroscedasticity and normality of the residuals were observed in both models through visual inspection and Cameron & Trivedi tests. These observations required a re-analysis of the two models using robust linear regression procedures as an alternative to least squares regression.

Spearman’s rank correlation was calculated to assess the relationship between alcohol consumption on one hand, and drinking debut age, DASS– 21R and SACS scores on the other. Also, the Mann-Whitney U test was used to evaluate gender differences regarding AC.

## Results

### Descriptive analysis

**Fig. 1:**
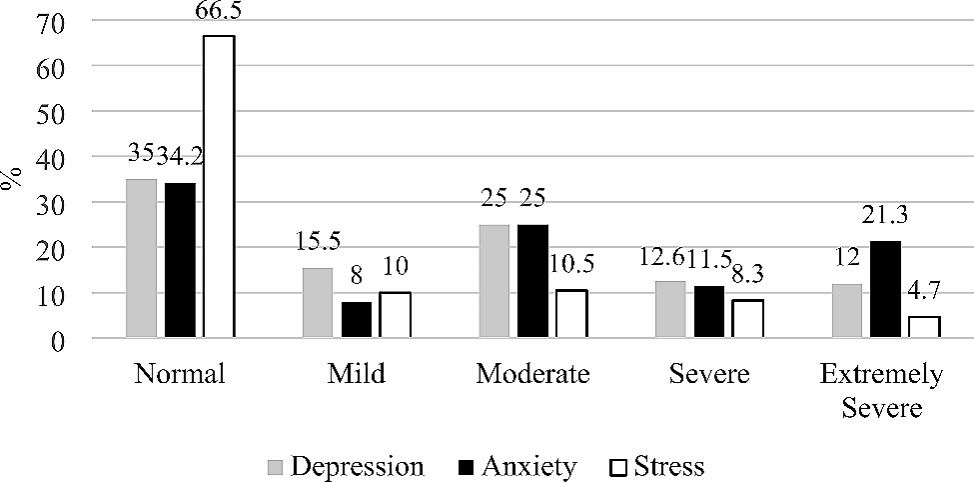
The proportion of scores for depression, anxiety and stress

The highest proportion of severe and extremely severe scores was observed for anxiety (32.8%) and depression (24.6%) (Fig. 1).

Among the highest scores, the largest weights were found for the following coping strategies: Instinctive action, Avoidance and Seeking social support. The highest weights for the lowest scores were recorded for the Antisocial action and Social Joining strategies (Table 1).

**Table 1:** Distribution of coping strategies by scores

The Strategic Coping Approach Scales (SCAS)	Low	Middle	High
Assertive action	17.5	64.9	17.5
Social Joining	**18.2**	64.6	17.2
Seeking social support	14.6	66.6	**18.9**
Cautious action	18.1	66.5	15.4
Instinctive action	14.1	65.9	**20.0**
Avoidance	16.6	64.4	**19.0**
Indirect action	15.4	70.0	14.5
Antisocial action	**20.9**	63.9	15.3
Aggressiveaction	15.3	68.5	16.2

The frequency of alcohol consumption in the analyzed sample according to the WHO classification is presented in Table 2.

**Table 2:** AUDIT classification

Category	Frequency	Percent
Low Risk (0–7)	592	87.4
Medium Risk (8–15)	69	10.2
Hazardous (16–19)	9	1.3
Extremely Hazardous (20+)	7	1.0
Total	677	100.0

After removing missing data, regression model one included 673 observations and indicated 5 predictors explaining about 15% of the variance in alcohol consumption (R2 = 0.147, F (5,667) = 22.93, *p* <.01). Age and caution action (Strategic Coping Approach Scale-SCAS) had an effect of lowering the alcohol consumption score. Anxiety, antisocial action and gender had an effect of increasing the alcohol consumption score.

After eliminating missing data, the second model included 667 observations and indicated that gender and 6 personality traits were found to have a significant relationship with alcohol consumption (R2 = 0.190, F(7,659) = 22.10, *p* <.01). Of these personality traits Health Concerns (GES), Life satisfaction (LEB), Social Orientation (SOZ) and Inhibitedness (GEH) had a lowering effect on the alcohol consumption score, while Aggressiveness (AGGR) and Somatic complaints (KORP) exerted an opposite effect. In both models, male subjects exhibited a higher alcohol consumption than female subjects. All VIF values were below the reference value of 10, indicating the absence of autocorrelations. The robust regression indicated that age was not a significant variable in the first model, and also the GES and SOZ personality traits were not significant variables in the second model. The final significant coefficients of bootstraped regression models are presented in Tables 3 and 4.

**Table 3:** Bootstrap for parameter estimates for model ONE

Variables	B	Bias	Std. Error	p	95%CI	
INTERCEPT	2.859	− 0.084	0.715	0.001	1.355	4.163
Caution Action	− 0.128	0.004	0.037	0.002	− 0.200	− 0.050
Antisocial Action	0.191	0.003	0.039	0.001	0.120	0.276
Anxiety	0.064	− 0.002	0.019	0.002	0.025	0.100

**Table 4:** Bootstrap for parameter estimates for model TWO

Variables	B	Bias	Std. Error	p	95%CI	
INTERCEPT	4.401	− 0.033	0.855	0.001	2.747	6.101
AGGR	0.333	0.005	0.059	0.001	0.222	0.450
LEB	− 0.242	0.001	0.082	0.004	− 0.407	− 0.084
GEH	− 0.215	0.002	0.049	0.001	− 0.311	− 0.111
KORP	0.148	− 0.001	0.068	0.029	0.009	0.269

Presenting negative B coefficients, caution action (from SCAS, B = -0.128, *p* =.002), Life Satisfaction LEB (from FPI-R, B = -0.242, *p* =.004), and Inhibitedness GEH (B = -215, *p* =.001) exerted a decreasing effect on the alcohol consumption score. A contrary effect was observed for anxiety (from DASS– 21R, B = 0.064, *p* =.002), antisocial action (from SCAS, B = 0.191, *p* =.001), and two personality traits (from FPI-R): Aggressiveness (AGGR, B = 0.333, *p* =.001) and Somatic complaints (KORP, B = 0.148, *p* =.029).

The Spearman rank correlation indicated that the AC score increases if an early onset is observed: ρ(661) = − 0.261, *p* <.01. The Spearman Test also identified significant but weak positive correlations between AC on one hand, and depression (ρ(661) = 0.146, *p* <.01), anxiety (ρ(661) = 0.147, p <. 01), stress (ρ(661) = 0.156, *p* <.01) (from DASS– 21R) and antisocial action (ρ(661) = 0.201, p <. 01) (from SACS) on the other hand. Significant weak negative correlations were observed between AC and assertive action (ρ(661) = − 0.101, *p* <.01) or cautious action (ρ(661) = − 0.129, *p* <.01) (from SACS).

Also, the Mann-Whitney U test indicated a significantly higher alcohol consumption in men (Mdn = 4.0) compared to women (Mdn = 2.0): U = 28464.0, z = -5.0, *p* <.01.

## Discussion

### AC vs. depression, anxiety and stress scales (DASS– 21R) and the strategic coping approach scale (SCAS)

Studies that addressed the consequences of the pandemic on mental health during its second wave indicated an increase of depressive and anxious symptoms [23].

As a result of the enforced isolation, during the second and third pandemic waves more than half of the students enrolled in the present research were affected by depression or anxiety of moderate to extremely severe intensity. Their weight was similar to that found in another study carried out on American youngsters [24] or to those observed in the meta-analysis of Oliveira Carvalho, Hülsdünker & Carson [25], yet lower than the one reported in an Indian study involving 235 students [26]. In the present study, as in other researches it was observed that anxiety affected subjects to a greater extent than depression [27].

The roots of anxiety and depression lay in the stress induced by disruptions of everyday life as a result of the prolongued pandemic. In this respect, the share of students reporting moderate to extremely severe stress was up to a quarter lower in our sample compared to the Indian study [26].

However, these prevalences may depend on other mediating factors such as safety, health concern [28], the presence of a sick family member, job insecurity or shortage of incomes [29]. In the sample of youth from the present study, significant but weak positive correlations were observed between AC and depression, anxiety, and stress.

Although for all coping mechanisms the highest weights (over 60%) were recorded at average level, it was observed that among those with high scores, Avoidance, Instinctive action and Seeking social support mechanisms were preferred.

In a sample of 996 Spanish subjects over 18 years of age, Muñoz-Violant et al. [30] found similar weights for high scores on the Avoidance coping mechanism. Also, avoidance was associated with more intense anxiety symptoms. So in this pandemic context it appears that Avoidance was a good mechanism rather than unprofitable as it is usually considered. It appears that Avoidance mechanisms were a positive solution in the pandemic context, as withdrawal and engaging in alternative activities rather that confronting exerted a protective influence.

When considering the younger generation, which was never faced with a crisis of such magnitude, activation of the Instinctive action coping mechanism implying an intuitive-impulsive-brisk approach to the problem is quite quite understandable. As our sample exhibited low cognitive availability towards uncertainty, the youngsters could have also activated the Seeking social support mechanism. Similarly, Klümper & Sürth, [31] have found that a perceived threat increased the need to seek information regarding the situation they were facing, as well as for social support. When finding themselves in problematic situations, people tend to seek support (emotional, instrumental) from family, friends, specialists or support groups. Most studies revealed an intensive social media usage in search for information [32], as well as an increased demand for psychotherapy via telepsychology [33] or assistance from virtual social support groups [34] during the pandemic.

As expected, our research highlighted low level scores in the Social Joining coping, prohibited by the restrictions imposed in order to prevent contamination with SARS-CoV-2. Low scores were also recorded for the Antisocial action mechanism. Alonso-Ferres et al. [35] concluded that personal exposure to adversities leads to pro-social responses. It can further be interpreted that the perceived health threat related to COVID-19 led to pro - rather than anti-social actions [36].

Significant but weak positive correlations were observed between AC and antisocial action (from SACS). As antisocial coping increases, AC also increases, so these can be considered factors that negatively potentiate each other.

Significant weak negative correlations were instead observed between AC and assertive action, cautious action (from SACS), AC increasing as assertive action and cautious action coping decreased. Therefore, assertive and avoidance coping can be considered protective factors against PAC.

### AC: prevalence, onset, gender

The prevalence of problematic alcohol consumption in the sample of young Romanian students was low: Hazardous and Extremely Hazardous (2.3) and Medium Risk (10.2).

It may be argued that students in the faculties of psychology, medicine, social work and biology, which this sample mainly consisted of, possessed better coping skills and stress reduction techniques compared to the general population, as Adler [37] found to be on social work students.

Rada & Lungu [14] found that the share of those with a low risk of AC according to the WHO Audit in a sample of 210 undergraduate and master’s students evaluated in the first three months of the pandemic was 83.8%. Of note, in the present sample it was 3.6% higher. Although the difference is not really large, it can be cautiously stated that the share of those with risky alcohol consumption decreased slightly, as Graupensperger, Calhoun, Fleming & Lee, [38] also found, probably as a consequence of the restrictions imposed by the pandemic (limited socialization, including time spent in clubs). In this sample no indicators of increased solitary alcohol consumption were observed.

Similar to pre-pandemic findings on AC [39, 40] the present study highlighted that an early AC onset increased the risk of problematic AC during the pandemic. It also revealed that the documented higher AC profile for males compared to females was preserved during the Pandemic in our student sample.

### AC vs. caution action (from SCAS) and life satisfaction LEB (from FPI-R)

The Caution action coping mechanism and high scores on the Life Satisfaction scale had the effect of decreasing the score on AC.

Caution action (from SCAS) is only apparently a passive strategy, and should not be confused with avoidance. Our findings were that it lowered the score for alcohol consumption in the pandemic context as this cautious strategy, implying a careful evaluation of one’s options for the benefit of safety and no later regrets, kept young people away from PAC drinking.

Taking precaution measures before acting, carefully evaluating all available options in order to avoid potential dangers, is a safe approach in general, more so in this particular context; the messages of scientists, physicians or international organizations during the COVID-19 pandemic emphasized that excessive alcohol consumption weakens the immune system, making it more susceptible to infection with the SARS-CoV-2 virus [13, 41, 42].

In a UK sample of 952 adult drinkers aged 18–79, Walker & de Visser [43] found that messages emphasizing the negative effects of alcohol on the immune system were a highly efficient tool for generating acceptance to safety recommendations during the Covid-19 lockdown. This sample, consisting mainly of subjects studying in health promoting fields, may have better understood the danger of excessive alcohol consumption. However, it is risky to conclude that this cautionary response to stress was conscious and deliberate rather than an automatic one. In this context of limited knowledge, precautionary action appeared similar to coping and solving problems even if the solution was obscure and no one could foresee the course of events.

This cyclic interdependence, in which alcohol abuse acted as a risk factor for the Covid-19 infection, while the Covid-19 pandemic was turning into a risk factor for alcohol abuse [44], was apparent to subjects which did not engage in excessive alcohol consumption in order to disengage from a stressor or simply feel better.

Subjects presenting high scores in Life satisfaction (LEB), with a high degree of self-acceptance, optimistic about their own future, sthenic, cheerful, i.e. those with a purposeful, positive life attitude and coherent achievement orientation, presented a lower risk of PAC. High scores on the LEB scale designate individuals with low excitability, few somatic complaints, and low neuroticism. In the present study, these characteristics were not associated with problematic alcohol consumption, which may suggest that these dimensions, as well as the life satisfaction personality dimensions, act as protective factors. This is consistent with the findings of Dymecka et al. [45] on a sample of 907 Polish subjects that health-related hardiness, a sense of coherence and life satisfaction during the COVID-19 pandemic were important resources for coping with difficult circumstances, thus outlining the relationship between sense of coherence and life satisfaction [46] According to Fahrenberg, Hampel & Selg, 2001 [20], the Life satisfaction scale correlates with Achievement orientation scale suggesting that ambitious, conscientious, thorough, and success-oriented individuals are at lower risk of engaging in excessive alcohol consumption.

The changed living conditions during the pandemic have generated uncertainty, anxiety and stress that could have led to problematic alcohol consumption as a means of relaxation and protection. The present study shows that young people with high life satisfaction, which approached the pandemic in a relaxed manner, were not exposed to excessive alcohol consumption.

### AC vs. anxiety (from DASS– 21R), antisocial action (from SCAS), aggressiveness (AGGR) and somatic complaints (KORP) (from FPI-R)

The Robust linear regression model indicated that Anxiety, Antisocial action (coping mechanism), Aggressiveness and Somatic complaints (personality traits) determined an increase of the AC scores. As previously mentioned, health concerns and support provided through virtual social networks led to the development of mutual help feelings, while isolation, restrictions or uncertainty generated increased violence at individual and collective level [47].

Secondary prevention (concerned with progression and relapse) and tertiary prevention (minimization of functional impairment) in regular and heavy drinkers have been widely studied. However, fewer studies have addressed the mediating factors that can elucidate the mechanisms underlying the association between stress and alcohol consumption [48]. A mediating factor addressed in this study as a personality trait is aggressiveness.In the present study, high scores on the aggression scale were associated with high scores on alcohol consumption. People who get angry easily, are choleric, labile, have low self-control or exhibit dominant reactive behaviors with a tendency to assert themselves, present a higher risk of PAC. According to the authors of FPI-R the Aggressiveness scale correlates with the Excitability one, i.e. persons with a high degree of activism, sometimes overreactive, are also susceptible to develop drinking problems. As a result, education for self-control and balanced behaviors is necessary. This aspect is all the more important since the majority of aggression acts are manifested against the background of alcohol consumption.

Probably this worldwide experience brings into question a reconfiguration of social psychology, namely the Social Psychology of aggression [49].

High scores on Somatic complains were associated with high scores on alcohol consumption. Asthenic, sickly, distrustful, withdrawn, pessimistic people, those who often exaggerate personal worries, were at risk for problematic alcohol consumption. The stress experienced during the pandemic was manifested by an increase in affective and somatic complaints [50, 51].

According to the authors of instrument the Somatic complaints scale correlates negatively with Life Satisfaction (LEB) and positively with the Inhibitedness (GEH), Excitability (ERR) and Strain (BEAN) scales. If these positive correlations are taken into account, it can be stated that the youngsters who would describe themselves as having a reactive behavior, low self-control, feelings of anger over long periods of time (High Excitability), those who were overloaded with work-related tasks, feeling exhausted and stressed (high Strain), were at risk of problematic alcohol consumption.

The results of this study regarding the increase in the alcohol consumption levels in those recording high scores on the Aggressiveness and Somatic complaints are consistent with the ones found in a literature review [52], namely that during the pandemic the mental health of people with dysfunctional personality traits were affected to a greater extent than that of those with positive personality traits.

Neuroticism (i.e. emotionality) as described by Eysenck is similar to the Excitability trait from the FPI-R questionnaire used in this study, sharing negative feelings such as anger, depression, anxiety or guilt. Freitag & Hofstetter [53] found that neuroticism was related to the perception of threats and emotional responses to the Covid-19 pandemic. Considering the students in the present sample, it was found that those emotionally stable, exerting control over their emotions, presented a lower risk of problematic alcohol consumption during the pandemic.

In the present research, youngsters reporting anxiety were at risk for AC. The result is similar to the findings of several other studies that people with high levels of anxiety related to COVID-19 were at greater risk of drinking in order to cope with a context characterized by the continued spread of the disease and prolonged isolation, resulting in a delayed return to university activities, lack of personal contact with colleagues and teachers, etc. [54, 55].. Before the pandemic, studies identified that some people turned to alcohol to overcome anxiety. The problem is more complex because a dangerous interaction occurs: coping with anxiety through alcohol consumption subsequently increases anxiety [56].

In regard to our sample, the use of the antisocial action coping mechanism i.e. approaching stressful situations by acting according to one’s personal needs regardless of any negative consequences on others, also increased the risk of AC. It is a complex association since problematic AC increases the risk of aggression, violence. Studies have shown increased susceptibility to aggression and antisocial behavior against the background of alcohol consumption [57].

This is where the problems raised by the antisocial personality, characterized by antisocial coping, come into question. The prolonged tension experienced during the pandemic led to negative emotions which diminished the ability to control antisocial behaviors. In a sample of in young people who had problems with the law, possibly some with antisocial disorder, Reid, Chenneville, Gardy&Baglivio [58] identified an increased aggressive behavior, troublesome in-school conduct and problems related to substance consumption.

### Limitations of the study

Although the results of this study are important because they bring attention to the psychological factors involved in alcohol consumption, it should be noted as a limitation that the sample was not representative of the population of young students and master’s students in Romania, including too few male subjects, from specializations with a technical profile, arts and theater and film.

## Conclusions

During the COVID-19 pandemic, after 3 months from the installation of the state of emergency on the territory of Romania, data were collected over a period of almost 18 months from students and master’s students enrolled at various universities. The main objective of this study was to explore the determinants of problematic alcohol. Three main categories of variables were taken into account: (a) emotional states of depression, anxiety and stress, (b) the behavioral dimensions of coping (c) personality traits. The influence of gender and onset of alcohol consumption on the risk of problematic alcohol consumption were also analyzed.

The hypothesis that emotional states, coping behavioral dimensions, personality traits, age and gender can be significant predictors for alcohol consumption was confirmed and the research questions were answered as follows.

- Early onset increases the subsequent risk of problematic AC.
- Compared to women, men recorded a higher AC.

Most respondents were studying in health-promoting fields, implying a better understanding of the negative effects of AC, more so during a pandemic when the immune system was already vulnerable and the knowledge of stress reduction techniques compared to the general population contributed to a low prevalence of problematic AC.

PAC decreased during the pandemic, probably as a result of the closure of entertainment venues. Occasional alcohol consumption in bars, restaurants or at home is generally a harmless way of relaxing and socializing. Education is essential in order to keep AC under control, preventing the development of alcohol use disorders leading to various social problems.

The risk factors for AC were psychological states, depression, anxiety (mainly), stress, antisocial coping mechanism and high scores on the Aggressiveness and Somatic complaints personality dimensions.

In contrast, appealing to assertive action and cautious action coping mechanisms, as well as high scores on the Life Satisfaction personality dimension, acted as protective factors.

This study advocates for the education of youngsters to clearly express their opinions without violating the boundaries of others’ feelings (assertive action) and to act prudently in dangerous or uncertain situations (cautious action) since these coping mechanisms were not associated with problematic alcohol consumption.

The promotion of positive, achievement-oriented, life attitudes is equally important, as these characteristics of the Life Satisfaction personality dimensions were also found as non-determinants of alcohol-induced problems.

Possessing low cognitive availability when confronted with a crisis of unprecedented magnitude, youngsters instinctively chose Avoidance, Instinctive action and Seeking social support as coping mechanisms. In a context of worldwide uncertainty induced by the pandemic it was natural that withdrawal, expectative atitudes and intuitive problem approaching appeared as protective solutions. When seeking social support to counter the perceived threats during lockdown, social networks and electronic communication were the easily accesible means. However, excessive media exposure inherently generated stress and anxiety.

The association of problematic alcohol consumption with antisocial actions as a coping mechanism identified in the present Romanian study calls for interventions to educate the younger generation how to acquire and adopt healthy mechanisms to control tensions without resorting to alcohol consumption.

Since high scores on Aggressiveness have been associated with problematic alcohol consumption, another focus in the educational approach is the education of self-control and behavioral balance, more so as the two variables reinforce each other.

Asthenic, overly anxious, pessimistic individuals were more vulnerable to problematic AC, probably resorting to drinking in order to change their negative affective mood. As this Somatic complaints scale correlates negatively with the Satisfaction with life scale, it would be useful to dynamize it towards goal orientation and reconciliation with one’s own person. Drinking as a means of gaining courage must be carefully reconsidered since anxiety generally hits back, often in increased levels.

## Data Availability

The database supporting the reported results is available only upon request due to confidentiality and ethical restrictions. This can be requested from the author of the correspondence.

## Abbreviations

PAC: problematic alcohol consumption
AC: alcohol consumption
DASS– 21R– 21R: Depression, Anxiety and Stress Scales
SCAS: Strategic Coping Approach Scale
FPI-R: Freiburg Personality Inventory

## References

[1] Killgore WDS, Cloonan SA, Taylor EC, Anlap I, Dailey NS (2021). Increasing Aggression during the COVID-19 lockdowns. J Affect Disorders Rep.

[2] Abreu L, Koebach A, Díaz O, Carleial S, Hoeffler A, Stojetz W et al. Life With Corona: Increased Gender Differences in Aggression and Depression Symptoms Due to the COVID-19 Pandemic Burden in Germany. Frontiers in Psychology [Internet]. 2021;12:689396. Available from: https://www.frontiersin.org/articles/10.3389/fpsyg.2021.689396. 10.3389/fpsyg.2021.689396 PMC8353131 34385959

[3] Bonea G-V, Buligescu B, Mihaiu S (2022). Domestic violence before and during the first year of the Covid-19 pandemic: a rapid review of the context in Romania. J Community Posit Practices.

[4] Al-Sejari MM, Al-Ma’seb HB (2022). Aggression and violence during the lockdown caused by the COVID-19 pandemic in Kuwait. J Affect Disorders Rep.

[5] Park AH, Zhong S, Yang H, Jeong J, Lee C. Impact of COVID-19 on physical activity: A rapid review. J Glob Health. 2022;12:05003. Available from: https://jogh.org/documents/2022/jogh-12-05003.pdf. 10.7189/jogh.12.05003 PMC8979477 35493780

[6] Rezapour M, Dehzangi A, Saadati F (2022). Students’ negative emotions and their rational and irrational behaviors during COVID-19 outbreak. PLoS ONE.

[7] Delpino FM, da Silva CN, Jerônimo JS, Mulling ES, da Cunha LL, Weymar MK, Alt R, Caputo EL, Feter N (2022). Prevalence of anxiety during the COVID-19 pandemic: a systematic review and meta-analysis of over 2 million people. J Affect Disord.

[8] Daly M, Robinson E (2022). Depression and anxiety during COVID-19. Lancet [Internet].

[9] O’Reilly A, Tibbs M, Booth A, Doyle E, McKeague B, Moore J. A rapid review investigating the potential impact of a pandemic on the mental health of young people aged 12–25 years. Irish Journal of Psychological Medicine. Volume 38. Cambridge University Press; 2021. pp. 192–207. 3https://pubmed.ncbi.nlm.nih.gov/32912358/. 10.1017/ipm.2020.106 PMC7711353 32912358

[10] Stroud I, Gutman LM (2021). Longitudinal changes in the mental health of UK young male and female adults during the COVID-19 pandemic. Psychiatry Res.

[11] Jaffe AE, Kumar SA, Hultgren BA, Smith-LeCavalier KN, Garcia TA, Canning JR (2022). Meaning in life and stress-related drinking: a multicohort study of college students during the COVID-19 pandemic. Addict Behav.

[12] Schecke H, Bohn A, Scherbaum N, Mette C (2022). Alcohol use during COVID-19 pandemic on the long run: findings from a longitudinal study in Germany. BMC Psychol.

[13] Calina D (2021). COVID-19 pandemic and alcohol consumption: impacts and interconnections. Toxicol Rep [Internet].

[14] Rada C, Lungu M (2023). The involvement of age, gender, and personality variables in Alcohol Consumption during the start of the COVID-19 pandemic in Romanian University students. Behav Sci.

[15] Cheng H, Wang D, Wang L, Zou H, Qu Y. Global prevalence of self-harm during the COVID-19 pandemic: a systematic review and meta-analysis. 2023;11(1):149–9. 10.1186/s40359-023-01181-8. 10.1186/s40359-023-01181-8 PMC10160734 37147683

[16] Ministerul, Justiției. Sept, Oficiul Național al Registrului Comertului. Legislaţie COVID19[ Ministry of Justice, National Trade Registry Office. COVID19 legislation]. https://www.onrc.ro/index.php/ro/legislatie/legislatie-covid19#Decrete. Accessed 1 2023.

[17] Lovibond SH, Lovibond PF. DASS: manual pentru scalele de depresie, anxietate şi stres: DASS 21-R, adaptat în România de Perțe, A. coord. Albu, M.; 2011. [DASS. Manual for the depression anxiety stress scales. 2nd ed. Sydney, N.S.W.: Psychology Foundation Of Australia; 1995. Adapted for Romania by Perțe, A. coord. Albu, M. DASS 21-R. Cluj-Napoca: ASCR Publishing House 2011].

[18] Budău O, Albu M. Scala de abordare strategică a copingului (SACS) [Strategic Coping Approach Scale, (SACS)]. Cluj-Napoca: Editura ASCR [Cluj-Napoca. ASCR Publishing House]; 2010.

[19] World Health Organization, Babor TF, Higgins-Biddle JC, Saunders JB, Monteiro MG, AUDIT.: The Alcohol Use Disorders Identification Test: Guidelines for Use in Primary Health Care, 2nd ed.; World Health Organization: Geneva, Switzerland, 2001. Available from: https://apps.who.int/iris/handle/10665/67205.

[20] Fahrenberg J, Hampel R, Selg H, Freiburger Personlichkeitsinventar. 2001, adaptat în România de Pitariu, H. P. și Iliescu, D. [Personality inventory Freiburg adapted for Romania by Pitariu, H. P. & Iliescu, D. - (FPI-R)]. Editura Sinapsis Publishing Projects, distribuit sub licență de DandD Consultantag Group, SRL, Testcentral [Sinapsis Publishing Projects, distributed under licence by DandD Consultantag Group, SRL, Testcentral]; 2015.

[21] IBM Corp (2019). Released. IBM SPSS statistics for Windows, Version 26.0. [Computer Software].

[22] StataCorp (2021). Stata Statistical Software: Release 18. [Computer Software].College Station.

[23] Matić T, Pregelj P, Sadikov A, Rus Prelog P, Depression (2022). Anxiety, stress, and suicidality levels in young adults increased two years into the COVID-19 pandemic. Int J Environ Res Public Health.

[24] Adams SH, Schaub JP, Nagata J, Park MJ, Brindis CD, Irwin CE. Young adult anxiety or Depressive Symptoms and Mental Health Service utilization during the COVID-19 pandemic. J Adolesc Health. 2022;70(6). 10.1016/j.jadohealth.2022.02.023. 10.1016/j.jadohealth.2022.02.023 PMC8995928 35422363

[25] Oliveira Carvalho P, Hülsdünker T, Carson F. The Impact of the COVID-19 Lockdown on European Students’ Negative Emotional Symptoms: A Systematic Review and Meta-Analysis. Behavioral Sciences [Internet]. 2022;12(1):3. Available from: https://www.mdpi.com/2076-328X/12/1/3. 10.3390/bs12010003 PMC8772797 35049614

[26] Verma H, Verma G, Kumar P (2021). Depression, anxiety, and stress during Times of COVID-19: an analysis of youngsters studying in Higher Education in India. Rev Socionetwork Strategies.

[27] Yau JTJ, Nager AL. Adolescent and young adult stress and coping during COVID-19: the utility of a pediatric emergency department screener. Int J Emerg Med. 2021;14(41). 10.1186/s12245-021-00359-4. 10.1186/s12245-021-00359-4 PMC8314256 34315406

[28] Werner AM, Tibubos AN, Mülder LM, Reichel JL, Schäfer M, Heller S (2021). The impact of lockdown stress and loneliness during the COVID-19 pandemic on mental health among university students in Germany. Sci Rep.

[29] Mohammadi F, Oshvandi K, Shamsaei F (2021). The mental health crises of the families of COVID-19 victims: a qualitative study. BMC Fam Pract.

[30] Muñoz-Violant S, Violant-Holz V, Gallego-Jiménez MG, Anguera MT, Rodríguez MJ (2021). Coping strategies patterns to buffer the psychological impact of the State of Emergency in Spain during the COVID-19 pandemic’s early months. Sci Rep.

[31] Klümper L, Sürth S. Keep me updated! Social support as a coping strategy to reduce the perceived threat caused by the cognitive availability of COVID-19 relevant information. Current Psychology. 2021; 1. 42(8)6403–6418. https://link.springer.com/article/10.1007/s12144-021-01951-w. 10.1007/s12144-021-01951-w PMC8205514 34149268

[32] DeFilippis E, Impink SM, Singell M, Polzer JT, Sadun R. The impact of COVID-19 on digital communication patterns. Humanit Social Sci Commun. 2022;9(1). 10.1057/s41599-022-01190-9.

[33] Sampaio M, Navarro Haro MV, De Sousa B, Vieira Melo W, Hoffman HG (2021). Therapists make the switch to Telepsychology to safely continue treating their patients during the COVID-19 pandemic. Virtual reality Telepsychology May be next. Front Virtual Real.

[34] Cordero DA (2021). Online support groups during the COVID-19 pandemic: a necessity or an added calamity. J Public Health.

[35] Alonso-Ferres M, Navarro-Carrillo G, Garrido-Macías M, Moreno-Bella E, Valor-Segura I. Connecting perceived economic threat and prosocial tendencies: The explanatory role of empathic concern. Capraro V, editor. PLOS ONE. 2020;15(5):e0232608. 10.1371/journal.pone.0232608. 10.1371/journal.pone.0232608 PMC7197816 32365125

[36] Serrano-Montilla C, Alonso-Ferres M, Navarro-Carrillo G, Lozano LM, Valor-Segura I (2021). Assessment of the effects of health and financial threat on prosocial and antisocial responses during the COVID-19 pandemic: the mediating role of empathic concern. Pers Indiv Differ.

[37] Adler D. Social worker student’s anxiety, and alcohol consumption during the Covid-19 pandemic. Electronic Theses, Projects, and Dissertations [Internet]. 2022 Jun 1 [cited 2023 Nov 2]; Available from: https://scholarworks.lib.csusb.edu/etd/1417.

[38] Graupensperger S, Calhoun BH, Fleming CB, Lee CM. Trends in Young Adult Alcohol and Cannabis Use through the First Year and a half of the COVID-19 pandemic from a community cohort sample. J Stud Alcohol Drugs 2023 Feb 22. 84(4), 489–98. 10.15288/jsad.22-00262. 10.15288/jsad.22-00262 PMC10488305 36971770

[39] Enstad F, Evans-Whipp T, Kjeldsen A, Toumbourou JW, von Soest T. Predicting hazardous drinking in late adolescence/young adulthood from early and excessive adolescent drinking - a longitudinal cross-national study of Norwegian and Australian adolescents. BMC Public Health. 2019;19(1). 10.1186/s12889-019-7099-0. 10.1186/s12889-019-7099-0 PMC6588913 31226962

[40] Aguilar MPO, Palomera PR, Núñez CL, Maroño CT, Gallegos SV, Cabrera NJP (2022). The role of age of Onset in Problematic Alcohol Consumption: artefact or cohort effect?. Clínica Y Salud.

[41] WHO. Alcohol Does Not Protect Against COVID-19; Access Should Be Restricted During Lockdown. [Online]. 2020. Available from: https://www.emro.who.int/mnh/news/alcohol-does-not-protect-against-covid-19-and-its-access-should-be-restricted-during-lock-down.html.

[42] Lebin JA, Mudan A, Wu AHB (2020). Chronic alcohol use does not protect against COVID-19 infection. Am J Emerg Med.

[43] Walker FC, de Visser RO. Messages focused on the effect of alcohol on the immune system boosted intention to adhere to alcohol intake guidelines during Covid-19 lockdown. Psychol Health. 2022;1–16. 10.1080/08870446.2022.2145606. 10.1080/08870446.2022.2145606 36412106

[44] Bantounou MA. A narrative review of the use of alcohol during the Covid-19 pandemic; effects and implications. J Addict Dis. 2022;1–11. 10.1080/10550887.2022.2058852. 10.1080/10550887.2022.2058852 35373718

[45] Dymecka J, Gerymski R, Machnik-Czerwik A, Derbis R, Bidzan M. Fear of COVID-19 and life satisfaction: the role of the Health-related hardiness and sense of coherence. Front Psychiatry. 2021;12. 10.3389/fpsyt.2021.712103. 10.3389/fpsyt.2021.712103 PMC8591072 34790135

[46] Moksnes UK, Løhre A, Espnes GA (2013). The association between sense of coherence and life satisfaction in adolescents. Qual Life Res.

[47] West SJ, Wood AM, Aboutanos MB, Thomson ND (2023). Exploring changes in violence across two waves of the COVID-19 pandemic in Richmond, VA. Aggressive Behav.

[48] Wittgens C, Muehlhan M, Kräplin A, Wolff M, Trautmann S (2022). Underlying mechanisms in the relationship between stress and alcohol consumption in regular and risky drinkers (MESA): methods and design of a randomized laboratory study. BMC Psychol.

[49] Krahé B (2020). The Social Psychology of Aggression. Routledge(3rd ed).

[50] Robillard R, Saad M, Edwards J, Solomonova E, Pennestri MH, Daros A, Veissière SPL, Quilty L, Dion K, Nixon A, Phillips J, Bhatla R, Spilg E, Godbout R, Yazji B, Rushton C, Gifford WA, Gautam M, Boafo A, Swartz R, Kendzerska T (2020). Social, financial and psychological stress during an emerging pandemic: observations from a population survey in the acute phase of COVID-19. BMJ Open.

[51] Chernetsova Y, Emelin V, Rasskazova E, Tkhostov A. Coping with emotions in pandemic as a factor of somatic complaints during lockdown. European Psychiatry. Volume 65. Cambridge University Press; 2022. pp. 490–S491. S110.1192/j.eurpsy.2022.1247.

[52] Di Stefano R, Di Pietro A, Talevi D, Rossi A, Socci V, Pacitti F, et al. Personality disorders (PD) and interpersonal violence (IV) during COVID-19 pandemic: a systematic review. Ann Gen Psychiatry. 2022;21(1). 10.1186/s12991-022-00388-0. 10.1186/s12991-022-00388-0 PMC8994418 35397587

[53] Freitag M, Hofstetter N. Pandemic personality: emotional reactions, political and social preferences across personality traits in times of Corona. Curr Psychol. 2021 Nov;23. 10.1007/s12144-021-02493-x. 10.1007/s12144-021-02493-x PMC8610108 34840485

[54] aan het Rot M, Baltariu IC, Enea V (2023). Increased alcohol use to cope with COVID-19-related anxiety one year into the coronavirus pandemic. Nordic Stud Alcohol Drugs.

[55] Fernandez MDS, Vieira IS, Silva NRJD, Cardoso TA, Bielavski CH, Rakovski C, Silva AER (2021). Anxiety symptoms and alcohol abuse during the COVID-19 pandemic: a cross-sectional study with Brazilian dental undergraduate students. J Dent Educ.

[56] Anker JJ, Kushner MG. Co-occurring Alcohol Use disorder and anxiety: bridging Psychiatric, Psychological, and neurobiological perspectives. Alcohol Res. 2019;40(1). 10.35946/arcr.v40.1.03. 10.35946/arcr.v40.1.03 PMC6927748 31886106

[57] Sontate KV, Rahim Kamaluddin M, Naina Mohamed I, Mohamed RMP, Shaikh MF, Kamal H, et al. Alcohol, aggression, and violence: from Public Health to Neuroscience. Front Psychol [Internet]. 2021;12(699726). 10.3389/fpsyg.2021.699726. 10.3389/fpsyg.2021.699726 PMC8729263 35002823

[58] Reid JA, Chenneville T, Gardy SM, Baglivio MT (2021). An exploratory study of COVID-19’s impact on psychological distress and antisocial behavior among justice-involved youth. Crime Delinquency.

